# Physicians' knowledge of health-related quality of life and perception of its importance in daily clinical practice

**DOI:** 10.1186/1477-7525-8-43

**Published:** 2010-04-23

**Authors:** Maurizio Bossola, Rita Murri, Graziano Onder, Adriana Turriziani, Massimo Fantoni, Luca Padua

**Affiliations:** 1 Department of Surgery, Catholic University of the Sacred Heart, Largo A Gemelli, 8 - 00168, Rome, Italy; 2 Institute of Infectious Diseases, Catholic University of the Sacred Heart, Largo A Gemelli, 8- 00168, Rome, Italy; 3 Department of Geriatrics, Catholic University of the Sacred Heart, Largo A Gemelli, 8 - 00168 Rome, Italy; 4 Department of Radiotherapy, Catholic University of the Sacred Heart, Largo A Gemelli, 8 - 00168 Rome, Italy; 5 Institute of Neurology; Catholic University of the Sacred Heart, Largo A Gemelli, 8- 00168, Rome, Italy; 6 Fondazione Don Gnocchi, Milan, Italy

## Abstract

**Background:**

Health-related quality of life (QoL) has become a crucial outcome in medical care. However, few studies have assessed physician knowledge of QoL and rate of physicians adopting QoL measures in clinical practice. The present study aimed at assessing the level of knowledge of QoL and the perceived importance of incorporating QoL assessment in clinical practice among physicians of a tertiary level academic hospital in Rome, Italy.

**Materials and methods:**

A survey study performed through the distribution of a questionnaire assessing knowledge of QoL studies that used the SF-36 scale, participation in studies evaluating QoL as well as knowledge of journals publishing articles on QoL Physicians and residents at the hospital Policlinico Gemelli, Catholic University of Rome.

**Results:**

Three-hundred nine physicians completed the questionnaire. Thirty-eight percent % reported knowing studies on QoL and using their results in clinical practice or for research purposes; 29% reported knowing the SF-36 questionnaire; 30% stated that at least one study assessing QoL had been conducted in their department. Fourty-six percent % stated that QoL must influence much or very much diagnostic choices and an even higher percentage reported that QoL must influence much or very much therapeutic and palliative strategies (70.8% and 91.3%, respectively). Reported barriers to the use of QoL measures in clinical practice were related to time constraints (8.7%) but also to doubts on methodological issues of QoL (30.7%). The large majority of physicians (94.3%) would have used more expensive drugs if these could improve QoL.

**Conclusions:**

The present study shows that in a tertiary level academic italian hospital one third of the physicians, reported to know QoL measures and that more than 80% of them would like to use QoL in their daily clinical practice. Future studies are needed to identify the best strategies to implement the use of QoL measures in clinical practice.

## Introduction

Health-related quality of life (QoL) has become a crucial outcome in medical care [1,2]. A communication style in which physicians ask their patients about both physical health problems and psycho-social issues has been found to be related to a higher patient satisfaction and even better health outcomes [3,4]. However, little is known about physicians' attitudes towards QoL and rate of physicians adopting QoL measures in clinical practice [5,6]. For example, a study exploring the knowledge of hospital physicians about QoL assessment revealed that less than two-thirds had some knowledge of QoL assessment in oncology [5]. Recently, a survey among oncologists has shown that there is lack of understanding of the justification and rationale for QoL assessment, lack of guidance on implementing assessment and limited knowledge of literature about QoL [6]. The present study aimed at assessing the level of knowledge of QoL and the perceived importance of incorporating QoL assessment in clinical practice among physicians of a tertiary level academic hospital in Rome, Italy.

## Methods

The survey was conducted on physicians and residents at the hospital Policlinico Gemelli, Catholic University of Rome. The Policlinico Gemelli is a 1700-bed academic hospital, located in the North of the town, built in 1964 (with more than 57.000 admissions per year). It is a public hospital and it is classified as hospital of a National relevance with high specialization. The mission of the hospital is to provide the best health care services, to implement a partnership with patients, to implement the best education in medicine and in management of health care services, and to combine technical health care services with a managerial approach. Since 1997 a process of re-engineering is ongoing with the purpose of developing a new vision of patient care, focused on patient centredness, on efficacy, effectiveness, and safety. The Policlinico Gemelli staff includes 999 physicians and 320 residents. Two hundred nine physicians (21.9%) were not included in the survey since they do not have contact with patients (for example microbiologists, laboratory personnel) The questionnaire items assessed knowledge of studies that used the SF-36 scale, participation in studies evaluating QoL and knowledge of journals publishing articles on QoL (Table 1). The SF-36 questionnaire was chosen since it is one of the most used instrument for the assessment of QoL in many different clinical fields.

**Table 1:** Questionnaire to assess the knowledge of QoL issue

What is your knowledge of QOL studies and trials?
- None
- Unspecific knowledge
- Specific knowledge without research activity
- Use of QOL assessment in clinical practice
- Use of QOL assessment for research purpose
**The SF-36 is:**
- Self administered and measures disability
- Self-administered, unspecific tool to measure QOL
- Filled by the physician
- Unknown

**Have you ever partecipated a study on QoL?**
- Never
- Once
- Several times

**Do you know a medical journal that publishes studies on QoL?**
- Yes
- No

**Studies on QoL are usually performed in your department?**
- Yes
- No

Similarly, the following attitudes of physicians towards QoL were also investigated: influence of QoL in diagnostic, therapeutic choices and palliative strategies; relevance of QoL outcomes in clinical studies and importance to plan QoL studies in the next future; role of physicians, nurses, psychologists in assessing QoL; barriers to the use of QoL in daily clinical practice (Table 2). Diagnostic strategies may have a different impact on QoL. For example, an accelerated diagnostic protocol for patients presenting to the emergency department with acute chest pain resulted in better QoL compared to the usual-care arm [7].

**Table 2:** Questionnaire to assess the perception of the importance of QoL in clinical practice

QoL must influence diagnostic strategies?
- No
- A little
- Quite
- Much
- Very much
- I do not know
**QoL must influence therapeutic strategies?**
- No
- A little'
- Quite
- Much
- Very much
- I do not know

**QoL must influence palliative care?**
- No
- A little'
- Quite
- Much
- Very much
- I do not know

**Do you have in mind QoL in your diagnostic and therapeutic strategies, although you don't meausre it?**
- No
- A little'
- Quite
- Much
- Very much

**When you read and score a scientific article is QoL important?**
- No
- A little'
- Quite
- Much
- Very much

**Is the measurement of QoL in clinical trials useful?**
- No
- A little
- Quite
- Much
- Very much

**Who should measure QoL?**
- Physician
- Other (nurse, psychologist)

**Is useful to increase the number of clinical trials in QoL?**
- No
- A little
- Quite
- Much
- Very much

**Should you use more expensive drugs if they shoul improbe QoL?**
- No
- A little
- Quite
- Much
- Very much

Physicians were also asked whether, among drugs with the same efficacy, they would have used those more expensive if these could improve QoL (Table 2). Five response categories were available from "no" to "very much".

The questionnaire was administered by mail, and a face-to-face distribution in every department was also done. For each department a reference person was identified to distribute and collect the filled questionnaire. Since the present study did not involve any patient and since all the data used for the analysis were provided directly by each participant, ethical approval was not requested.

### Statistical analysis

A descriptive analysis was performed. Moreover, to compare rate of answers among groups contingency tables were done and chi-square test applied. P < 0.05 was considered as statistically significant.

## Results

Out of 780 physicians and 320 residents, 308 (28%) completed the questionnaire. Their characteristics are shown in Table 3. One hundred sixty three (52.9%) were male. Forty-nine percent were < 35 years, 44.1% between 35-54 years, and only 7% were 55 years or older. Most (64.5%) worked in medical departments, 18.4% in surgical departments and 14.5% in radiology or in other services. The majority of responders answered to work in team. Considering the whole population of 999 physicians of the Gemelli Hospital, 68.4% are males, 27% less than 40 years-old, 44.6% more than 50 years-old; 395 (39.5%) work in medical areas.

**Table 3:** Characteristics of the physicians who answered the questionnaire

	**N (%)**
Gender:	
- Male	163(52.8)
- Female	146 (47.2)
Academic role:	
- Chairman/Associate Professor	38 (12.3)
- Asssistant Professor	82 (26.6)
- Assistano	63 (20.5)
- Residents	94 (30.5)
- Other	31 (10.1)
	
Tipe of activity	
- Within a team	256 (82.6))
- alone	37 (11.9)
- unknown	17 (5.5)
Age (years):	
- <34	149 (48.1)
- 35-44	71 (22.9)
- 45-54	66 (21.3)
- 55-64	22 (7.1)
- >65	1 (0.3)
Department	
- Medicine	200 (64.5)
- Surgery	57 (18.4)
- *Specialised Surgery*	*28 (49.1)*
- *General Surgery*	*29 (50.9)*
- Radiology- Nuclear Medicine - Other Services	45 (14.5)
- Unknown	8 (2.6)

As shown in Table 4, among the physicians who completed the questionnaire, 38% reported knowing studies on QoL and using their results in clinical practice or for research purposes; 29% reported knowing the SF-36 questionnaire; 30% stated that at least one study assessing QoL had been conducted in their department. Twenty three percent of physicians reported to have participated in studies assessing QoL whereas only 16.5% of them indicated the name of a journal publishing studies on QoL.

**Table 4:** Answers to the questionnaire assessing the knowledge of QoL issue

**What is your knowledge of QOL studies and trials?**	
None	48 (15.5)
- Unspecific knowledge	173 (55.8)
- Specific knowledge without research activity	31 (10)
- Use of QOL assessment in clinical practice	22 (7.1)
- Use of QOL assessment for research purpose	34 (11)
**The SF-36 is:**	
- Self administered and measures disability	15 (4.8)
- Self-administered, unspecific tool to measure QOL	90 (29)
- Filled by the physician	6 (1.9)
- Unknown	197 (63.5)
**Have you ever partecipated a study on QoL?**	
- Never	235 (75.8)
- Once	42 (13.5)
- Several times	32 (10.3)
**Do you know a medical journal that publishes studies on QoL?**	
- Yes	51 (16.5)
- No	259 (83.5)
**Studies on QoL are usually performed in your department?**	
- Yes	94 (30.3)
- No	216 (69.7)

However, as shown in Table 5, 46.1% of the physicians who completed the questionnaire stated that QoL must influence much or very much diagnostic choices and an even higher percentage of them reported that QoL must influence much or very much therapeutic and palliative strategies (70.8% and 91.3%, respectively). Seventy eight percent of physicians considered mandatory to measure QoL in clinical trials and to increase the number of QoL studies (67.4%). Most physicians (73.5%) reported that themselves or residents should measure QoL. The majority of physicians (94.3%) would have used more expensive drugs if these could improve QoL.

**Table 5:** Answers to the questionnaire assessing the perception of the importance of QoL in clinical practice

**Must QoL influence diagnostic strategies?**	
- No	15 (4.8)
- A little	46 (14.8)
- Quite	101 (32.6)
- Much	119 (38.4)
- Very much	24 (7.7)
- I do not know	4 (1.3)
**Must QoL influence therapeutic strategies?**	
- No	8 (2.6)
- A little	12 (3.9)
- Quite	66 (21.3)
- Much	166 (53.5)
- Very much	52 (16.8)
- I do not know	4 (1.3)
**Must QoL influence palliative care?**	
- No	3 (1)
- A little	2 (0.6)
- Quite	18 (5.8)
- Much	97 (31.3)
- Very much	186 (60)
- I do not know	3 (1)
**Do you have in mind QoL in your diagnostic and therapeutic strategies, although you don't meausre it?**	
- No	3 (1)
- A little	23 (7.4)
- Quite	121 (39)
- Much	130 (41.9)
- Very much	29 (9.4)
**When you read and score a scientific article is QoL important?** ?	
- No	38 (12.3)
- A little	70 (22.6)
- Quite	136 (43.9)
- Much	54 (17.4)
- Very much	6 (1.9)
**Is the measurement of QoL in clinical trials useful?**	
- No	1 (0.3)
- A little	16 (52)
- Quite	83 (26.8)
- Much	174 (56.1)
- Very much	35 (11.3)
**Who should measure QoL?**	
- Physician	228 (73.5)
- Other (nurse, psychologist)	73 (23.5)
**Is useful to increase the number of clinical trials in QoL?**	
- No	1 (0.3)
- A little	11 (3.5)
- Quite	54 (17.4)
- Much	190 (61.3)
- Very much	51 (16.5)
**Should you use more expensive drugs if they shoul improbe QoL?**	
- No	2 (0.6)
- A little	15 (4.8)
- Quite	68 (21.9)
- Much	167 (53.9)
- Very much	55 (17.7)

Reported barriers to the use of QoL measures in clinical practice were related to time constraints (8.7%) but also to doubts on methodological issues (30.7%).

We did not find any significant difference in the characteristics of the physicians who answered to know or not to know the SF-36 questionnaire. Results are shown in Table 6.

**Table 6:** Comparison between physicians who answered to know or not to know the SF-36 questionnaire

	Physicians who know SF-36 (N. = 89)	Physician who do not know SF-36 (N. = 215)	p
Female	46 (51.7)	98 (45.6)	
male	43 (48.3)	117 (54.4)	0.33
Academic role:			
- Professor/Associate professor	6 (6.7)	31 (14.4)	
- Assistant Professor	47 (52.8)	96 (44.7)	
- Resident	26 (29.2)	67 (31.2)	0.39
- Other	10 (11.2)	21 (9.8)	
Age			
- < = 44 years	68 (76.4)	147 (68.7)	0.18
- >44 years	21 (23.6)	67 (31.3)	
Medical area:			
- Medicine	66 (75)	132 (62.9)	
- Surgery	10 (11.4)	46 (21.9)	0.07
- Diagnostic/Service	12 (13.6)	32 (15.2)	
Did you partecipate studies on QoL?			
- yes	43 (48.9)	30 (13.9)	<0.0001
- no	45 (51.1)	186 (86.1)	

## Discussion

The present study shows that the majority of physicians who answered to the questionnaire on QoL are aware of its usefulness in clinical management. More than 80% of participants would like to use QoL in their daily clinical practice but only one third of the physicians who participated to the survey know QoL measures. To our knowledge, this is the first survey that on physicians of different specialities of a tertiary level academic hospital to evaluate their knowledge and perception of QoL.

Indeed, little is known about physicians' attitudes towards QoL and rate of physicians adopting QoL measures in clinical practice. An Italian study exploring the knowledge of hospital physicians on QoL assessment revealed that 62% had some knowledge of QoL assessment in oncology but that most tended to rely on a physician-base assessment rather than patient-based instruments [8]. In 1998, a survey of family physicians reported that for 78% of them it was possible to measure QoL, that 89% believed that QoL issue should be discussed with patients and that 89% would use a validated QoL measure if one were devised [9]. Meanwhile, Bezjak et al [10] collected information from a group of oncologists of a large Canadian cancer care centre on their perspectives on QoL and QoL information, through a self-administered questionnaire containing 75 items with a 4-point Likert categorical response scale. Of 67 eligible respondents, 54 replied. A total of 87% felt that published QoL data are useful for individual patient care, but 69% indicated that, at present, they would be more likely to base their recommendations on personal experience rather than on published literature and 57% felt that decisions were made more difficult when QoL issues were considered. Padua et al [11] conducted a fact-finding study among Italian neurologists to evaluate the degree of knowledge in the QoL field. Most responders indicated that it would be important either to increase knowledge of the real impact of a disease on a patient's QoL or to better evaluate the effects of therapy. More recently, Skevington et al. [12] approached 800 general practitioners in UK through the national postal system to find out if they used quality of life information in primary care, to explore their reasoning and to assess any barriers to use. Two hundred eighty physicians (38%) provided qualitative and quantitative information. The majority said that QoL was interesting and important. Users had seen more information and scales, and were more aware of their use; only 8% had ever used formal standardised questionnaires. The main barriers to implementation were a shortage of time and information, and experience with QoL assessment. A sizable minority wanted to know more. Seventy-one percent would use QoL to monitor treatment effectiveness.

Results from the present survey suggested that the importance of assessment of QoL is highly perceived. Many institutions are implementing programs including the assessment of patient-reported outcomes and, particularly, of QoL. The National Health Service (NHS) in Great Britain, for example, asks all patients who are having hip or knee replacements, varicose vein surgery or groin hernia surgery to fill in a questionnaire on patient-reported outcomes to help improving the quality of care. http://www.nhs.uk/NHSEngland/thenhs/records/proms/Pages/aboutproms.aspx. The gap between the knowledge of QoL and the perception of its importance documented in the present study merits further attention especially in order to find strategies to reduce this gap. Several causes may explain the gap: first, QoL methodology is scarcely taught during education courses [5]; second, papers including or primarily focusing on QoL are still rarely published in medical journals other than oncology journals [6]. Moreover, physicians caring for acute patients may pay a greater attention to survival rather than to QoL. We warrant that further studies may demonstrate whether education courses or spread of guidelines on QoL methodology for students, residents and physicians would result in decreasing the gap between perception of importance and feasibility of the QoL methodology in daily routine clinical practice and clinical trials.

Another interesting finding of this study is that, comparing the characteristics of physicians who answered to know or not to know the SF-36, we did not find significant differences. It seems that the knowledge of QoL measures is independent of age, gender, academic role, and medical speciality.

Also interesting is the finding that to the question *Who should measure the quality of life? *most answered that physicians should do so. We think that this is a topical issue. Some authors believe that physicians' perceptions of QoL may be at odds with those held by the patients and QoL assessment should be performed by the patients themselves using adequate and valid measures or, in alternative, by someone who acts as a proxy or surrogate, such as a family member or a health professional [13]. However, it is well known that many patients are unable to assess their own QoL and complete a QoL measure because of cognitive impairment, difficulties in communication, symptom-related distress, or complexity of QoL measure. Moreover, the high percentage of physicians answering they should personally measure QoL highlights the perception of the great importance of the QoL issue.

This study has several limitations. First, less than one fourth of the physicians answered the questionnaire and we do not know why about 70% of the physicians did not answer. It could be that they were not interested in the target of the study and in this case the conclusion of the study could be different. However, it is useful to underline that the rate of answer in surveys involving a large number of physicians may be low or very low [14]. Second, this is the result of one single Academic Center and findings can not be generalized to other settings. Third, since the questionnaire was built specifically for the purpose of the study, it was not previously validated. Finally, the meaning for some questionnaire items (for example for knowledge) can be differently interpretated by participants. However, the simplicity of the questions may have reduced the bias of variability in interpretation.

In summary, the present study shows that, in a tertiary level academic Italian hospital, one third of the physicians who answered to a questionnaire on QoL, reported to know QoL measures and over 80% of them would like to use QoL in their daily clinical practice. Further studies, also in different medical settings, could assess whether increasing the knowledge on health-related QoL could lead to a growing use of QoL measures in daily clinical practice, possibly increasing also the quality of clinical care.

## Competing interests

The authors declare that they have no competing interests.

## Authors' contributions

MB and RM designed and conducted the study as well as participated in the writing of the manuscript and in the statistical analysis. LP, MF, AT and GO contributed to the design of the study and to the statistical analysis. MF contributed to the writing and the revision of the manuscript. All authors read and approved the final manuscript.
